# Applying a New Automated Perimetry Pattern Based on the Stimulus Distribution of the Multifocal ERG to Improve Structure-Function Investigation in Glaucoma

**DOI:** 10.1155/2017/8780934

**Published:** 2017-11-07

**Authors:** Lívia M. Brandão, Matthias Monhart, Andreas Schötzau, Anna A. Ledolter, Anja M. Palmowski-Wolfe

**Affiliations:** ^1^Ophthalmology, Basel University Hospital, Basel, BS, Switzerland; ^2^Carl Zeiss AG, Feldbach, Switzerland; ^3^Department of Ophthalmology, Medical University of Vienna, Vienna, Austria

## Abstract

**Purpose:**

To validate a new automated perimetry pattern (mf103 pattern) for the investigation of retinal structure-function relationships in glaucoma in comparison to the standard G2 pattern and to relate either field's performance to optical coherence tomography (OCT).

**Methods:**

Automated perimetry data from the mfERG103 pattern were compared with the standard G2 pattern in glaucoma patients (18) and controls (15). The results of both (mean defect (MD) and mean sensitivity (MS)) were compared with optical coherence tomography (OCT): retinal nerve fiber layer (RNFL) thickness, macular thickness (mT), and ganglion cell analysis (GCIPL). Nine patients were followed up after one year.

**Results:**

G2 pattern and mf103 pattern did not differ significantly in MD or MS. The mf103 pattern associated significantly with more RNFL sectors in both MD and MS (*p* < 0.01 and *p* < 0.05, resp.). GCIPL thickness was not significantly associated with either SAP protocols. Both protocols remained comparable after one-year follow-up.

**Conclusions:**

G2 and mf103 pattern can both differentiate patients from controls with no significant difference in performance. RNFL thickness defects correlated better with mf103 than G2 with POAG. The mfERG-103 perimetry pattern can be used to establish structure-function correlations in glaucoma and may enable a more direct comparison with objective electrophysiological data.

## 1. Introduction

Glaucoma continues to be one of the leading causes of blindness worldwide [1], and structure-function correlations are currently investigated in an attempt to optimize performance [2–7]. This is important, for example, in some patients, standard automated perimetry (SAP) and, in others, optical coherence tomography (OCT) may be the first indicator of disease [8, 9]. Thus, there is still not a single examination which can provide precise and definitive early diagnosis [10].

In spite of evidence that standard automated perimetry reveals abnormalities only after a significant amount of ganglion cells has been lost [8, 11], it is still largely used as the “gold standard” for glaucoma diagnosis and follow-up. Recently, alternative electrophysiological methods have been proposed as an alternative objective measurement of glaucomatous retinal dysfunction, such as the pattern ERG [12–14] and the multifocal electroretinogram (mfERG) [15–21]. Hood et al. [22] have attempted to improve the correlation between visual field examinations and the mfERG in glaucoma by implementing a visual field (Humphrey) based on the mfERG and found poor correlation between visual field sensitivity and the standard mfERG [23]. Since then, recent applications of the mfERG in glaucoma have successfully focused on augmenting the inner retinal contribution to the mfERG to increase its sensitivity by, for example, including global flash paradigms [15–21, 24, 25].

In order to facilitate structure-function analysis and to allow better comparison between a multifocal ERG paradigm (2-global flash mfERG which presents global flashes between the m-sequence stimulation) [16, 25–27] and SAP in future studies, we customized an automated perimetry pattern (mf103 pattern, Octopus, Haag-Streit) to directly relate to the 103 mfERG stimulus grid. The perimetry results from the mf103 pattern were compared with those obtained using the G2 pattern. The results of both SAP methods were then compared to OCT findings in POAG and controls. In addition, nine patients were followed up after one year.

## 2. Materials and Methods

Eighteen POAG patients were recruited at the Glaucoma Service, Department of Ophthalmology, University of Basel. Additionally, 15 controls were recruited outside the clinical environment. Each subject underwent testing of best-corrected visual acuity (BCVA), Goldmann applanation tonometry, slit-lamp examination of the anterior segment, fundus biomicroscopy, automated perimetry (both protocols), and OCT. All examinations were performed by one of the authors (LMB). Informed consent was obtained prior to inclusion in the study. The study followed the tenets of the Declaration of Helsinki and was approved by the regional Ethics Committee.

Inclusion criteria for patients were the presence of a glaucomatous optic disc associated with thinning in the neuroretinal rim of the RNFL in the OCT and a normal intraocular pressure, if needed, controlled by topical medication.

Individuals were excluded from the study in cases of systemic diseases such as diabetes or arterial hypertension, if they had had any type of ocular surgery previously, if they were using medication which could influence the normal physiology of the eye, or if refraction was greater than plus/minus 6 diopters.

All patients were asked to return after one year, but only nine patients agreed to repeat both SAP protocols and OCT.

### 2.1. Automated Perimetry

Patients were tested with the standard glaucoma G2 program and the customized mf103 pattern in Octopus 900 (Haag-Streit AG, Switzerland). Overall MD and mean sensitivity (MS) were calculated directly by the machine software and compared between the two protocols. For structure-function analysis, focal values were exported and clustered offline to create averages corresponding to the specific areas examined in the OCT. Reliability was ensured by including only examinations with a fixation loss under 33% as well as false-positive and false-negative rates under 25%.

### 2.2. Automated Perimetry—Customized mf103 Pattern

**Figure 1:**
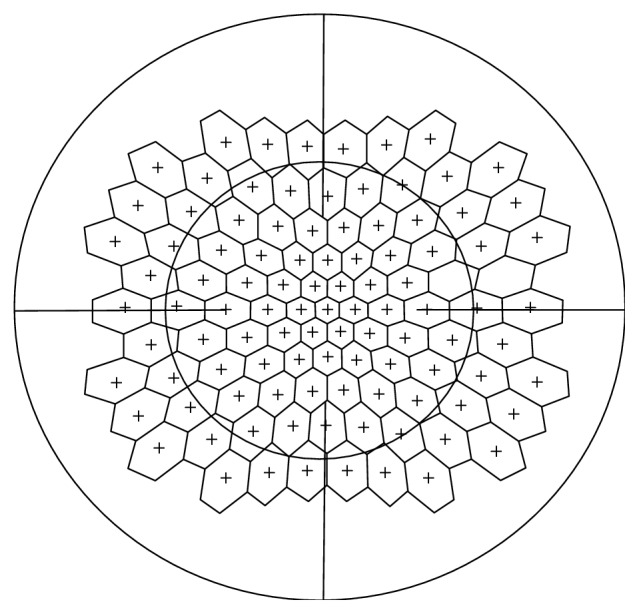
Superposition of the mf103 pattern and the mfERG stimulus grid (103 hexagons). The protocol has been developed to stimulate the individual areas, covering 50°, using the same test parameters as in standard perimetry testing: size III/3e; 100 ms duration; background 10 cd/m2; 0 dB equaled to 4000 asb.

The mf103 pattern was customized based on the stimulus distribution of the 103 multifocal ERG (Figure 1). Coordinates were calculated by one of the authors (MM) so each visual field light stimulus would correspond exactly to the center of each hexagon from the mfERG stimulus grid, resulting in 103 stimuli covering the central 50° of the retina. The mf103 pattern was tested as standard automated perimetry using a dynamic strategy with a white on white stimulus and was incorporated in the Octopus Perimeter 900. Thus, other than the different pattern of stimulation points, the same parameters applied as to the traditional glaucoma G2 protocol: stimuli size III, 100 ms duration, background 10 cd/m2, 0 dB equaled to 4000 asb. Using the original Octopus dataset, the software was able to calculate all commonly used parameters (MD, MS, and sLV) for the specific points of the mf103 pattern, which will be referred to as MD103, MS103, and sLV103.

### 2.3. OCT

Images from the optic disc and the macula were obtained with the spectral optical coherence tomography (Cirrus SD-OCT™) using macular cube (512 × 128) and optic disc cube (200 × 200) protocols. Total macula thickness (mT), ganglion cell-inner plexiform layer (GCIPL), and retinal nerve fiber layer (RNFL) thicknesses were calculated as per Cirrus software.

### 2.4. Structure-Function Analysis

**Figure 2:**
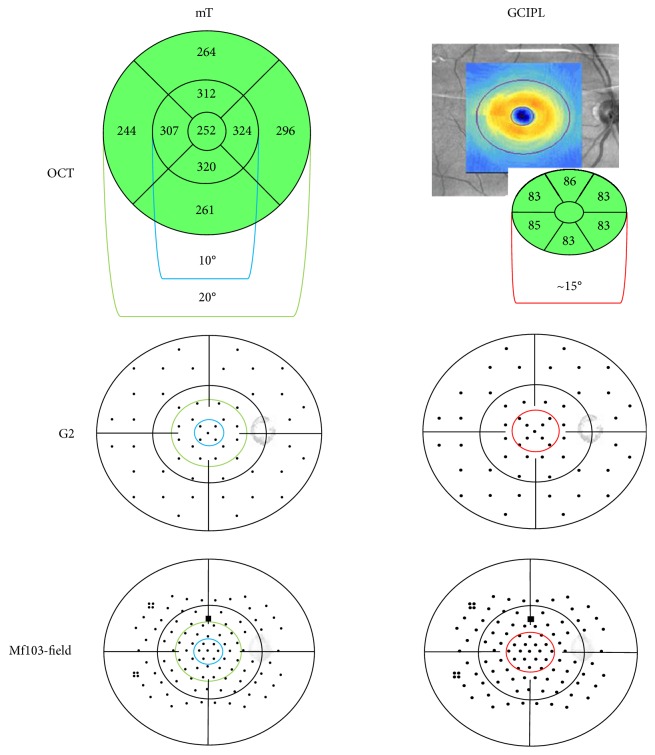
The group averages formed to compare OCT to visual field. The left column depicts the comparison between visual field and macular thickness, where the central 10° (mT10, 0–5° eccentricity) and 20° (mT20, 0–10° eccentricity) were analyzed. The right column displays the comparison between visual field and the GCIPL in the central ~15° (GCIPL, 0–7.5° eccentricity).

For structure-function investigation, visual field stimulus points were clustered according to the mT and GCIPL areas of the OCT (Figure 2). For comparison to macular thickness, we analyzed the central 10° (mT10, 0–5° eccentricity) and 20° (mT20, 0–10° eccentricity) and for comparison to the GCIPL, the central ~15° (GCIPL, 0–7.5° eccentricity). Clustering of visual field points took into account the displacement of the retinal ganglion cells in the central 10 degrees, as described by Drasdo et al. [28] and by Hood et al. [29]. Therefore, mT values for the central 20° and 10° corresponded, respectively, to the central 17 and 5 points in G2 pattern and 31 points and 7 points in the mf103 pattern protocol pattern. For comparison to GCIPL thickness measured within the central 15°, responses were averaged from the 9 central points of the G2 and the 19 central points from the mf103 pattern.

**Figure 3:**
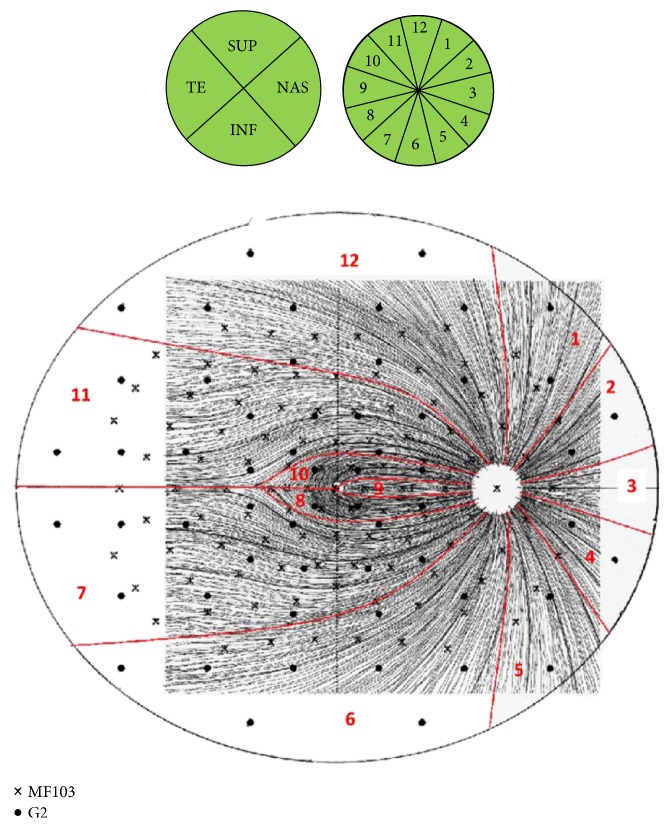
The top row shows the RNFL distribution maps from the Cirrus™ RNFL thickness maps, for quadrants (left) and clock-hour sectors (right). The lower graph is an overlay of the G2 pattern (black dots) and the mf103 patterns (x) onto the RNFL distribution map. Red lines separate each of the 12 group averages formed to relate to the respective RNFL clock-hour sectors. G2 and mf103 group clusters are based on original RNFL and Octopus correlation maps created by Bürki and Monhart [30]. Sup: superior; NAS: nasal; INF: inferior; TE: temporal; 1 to 12: individual clock-hour sectors; “**x**” mf103; and “•”: G2 pattern stimulus location.

RNFL measurements from the optic disc are expressed in Cirrus analysis as 4 sectors (superior, inferior, temporal, and nasal) and 12-clock-hour sectors (Figure 3, top). For comparison to the RNFL sectors, focal visual field values were clustered into 12 corresponding group averages (retinal view), taking into account the nerve fiber distribution and its relation to Octopus stimulus points as published by Bürki and Monhart [30] (Figure 3, bottom).

Demographic data from all 33 participants are described in Table 1.  Table 2 shows all OCT measures from both groups (mT, GCIPL, and RNFL).

### 2.5. Statistical Analysis

Demographic statistics were performed in SPSS (IBM, version 22). The statistical package R (version 3.0.2) was used to analyze the relationship between exams using linear mixed effects models (adjusted to age and gender) and to compare the areas under the receiver operating characteristic curves (AUC). Results are presented as slope coefficients with corresponding *p* values.

## 3. Results

On average, the G2 field testing required 5.35 minutes and the mf103 required 8.45 minutes. Thus, the mf103 pattern test was three minutes longer than the G2 pattern test. This was the same for glaucoma and control subjects. The specificity of both test patterns was 94.5%. On average, the G2 pattern flagged 18.8% which is 11 of 59 test locations as abnormal. The mf103 pattern flagged 22.0% which is 23 of the 103 locations as abnormal. Therefore, the mf103 test showed a slightly higher sensitivity at the same specificity as compared to the G2 pattern.

Table 3 shows that overall MD and MS from both visual field protocols were able to significantly differentiate glaucoma from controls (*p* < 0.01). G2 and mf103 pattern did not differ significantly in overall MD, MS, or sLV.

**Figure 4:**
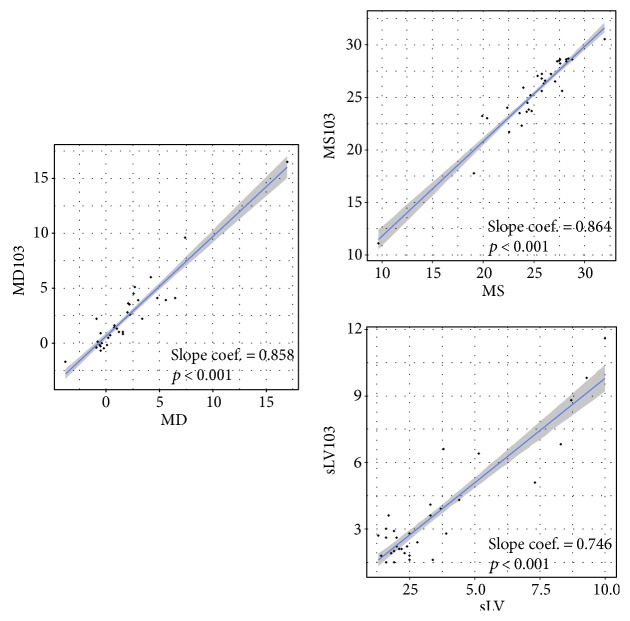
Linear mixed effects plots showing the significant positive relationship between G2 and mf103 patterns. For the G2 pattern, MD: mean defect; MS: mean sensitivity; and sLV: squared loss of variance; for the mf103 pattern, MD, MS, and sLV are marked with 103. All values are in dB.

Figure 4 shows a scatterplot of both visual fields depicting the significant positive relationship between both protocols for all parameters. When the performance was analyzed with the ROC curves, the AUC values did not differ significantly (DeLong test, Table 4).

**Figure 5:**
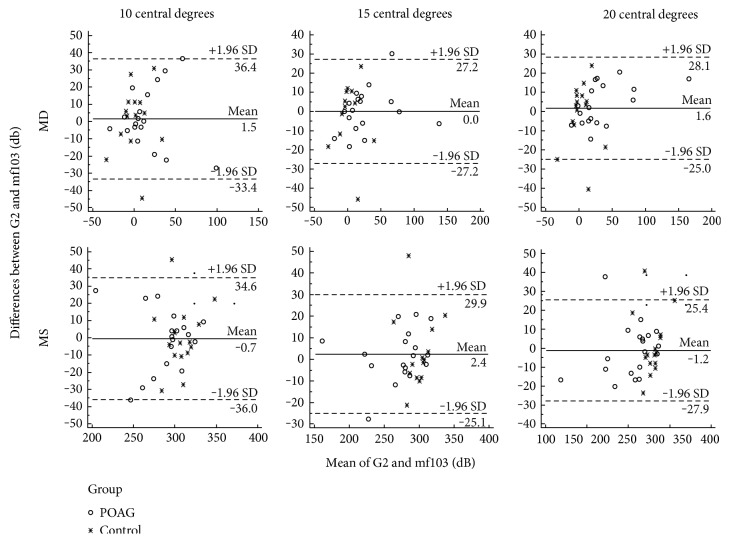
Bland-Altman plots (difference against mean) from MD and MS from the two different field patterns (G2 and mf103 pattern) for the central 10°, 15°, and 20°. MD: mean defect; MS: mean sensitivity; G2: standard stimulus pattern form; mf103: customized mf103 pattern; both in Octopus Automated Perimetry; POAG: primary open-angle glaucoma group; SD: standard deviation.

In the central 10°, MD or MS did not differ significantly between POAG and control. Consistently, no patients had field defects (≥3 adjacent points, *p* < 0.5%) in the central 10° in either G2 or the mf103 pattern. In the central 15° and 20°, MD was higher and MS was lower in POAG (*p* < 0.05). Three patients (16.6%) had a defect in G2 inside the central 15°, while 4 (22.2%) had a defect in mf103 pattern. For the central 20°, both visual field protocols identified 6 patients (33.3%) with a defect.  Figure 5 shows the Bland-Altman plots, that is, the difference against their mean for the two different field patterns and for the central 10°, 15°, and 20°. The mean difference was close to zero with no evidence of bias. Furthermore, the shallow defects seen in our early glaucoma patients can be appreciated as there is a large overlap with controls at these eccentricities.

### 3.1. Structure-Function Analysis

#### 3.1.1. RNFL × SAP

**Figure 6:**
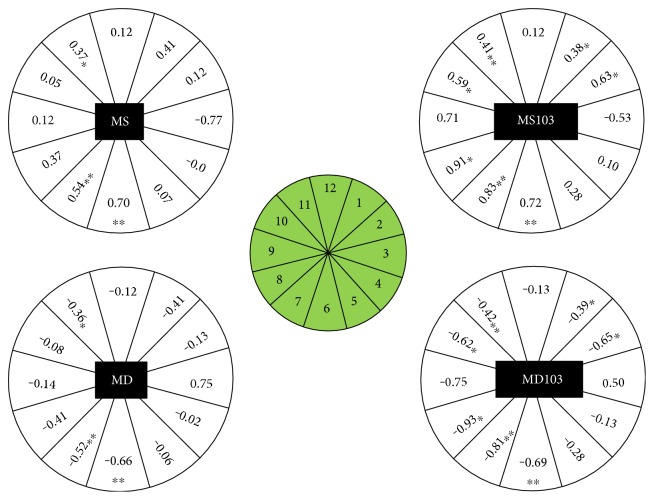
RNFL relationship to the G2 pattern (left) and to the mf103 pattern (right) is shown for the individual clock-hour sector (1 to 12). Numeric values are the regression coefficients from the linear mixed effects analysis. The level of significance is depicted by a “^∗^” (^∗^*p* < 0.01 and ^∗∗^*p* < 0.001). For the G2 pattern, MD: mean defect; MS: mean sensitivity; for the mf103 pattern, MD and MS are marked with 103.

The mf103 pattern protocol had a significant association with more RNFL clusters than G2 for MD (negative) and MS (positive), in the quadrant sectors (Table 5) as well as in the clock-hour sectors (Figure 6). While there was agreement in the inferior quadrant, more superior and temporal sectors of the mf103 pattern showed a significant positive (MS103) and negative (MD103) association with RNFL thinning when compared to G2.

#### 3.1.2. Macular Structure × SAP

In control subjects, neither mT nor GCIPL was significantly associated with G2 or with mf103 pattern MD or MS within the central 20° (10°, 15°, and 20°).

In POAG in the central 10°, the only significant association was between mT and MS (negative) as well as MD (positive) of G2 (*p* < 0.004). There was no significant association between mT and MS or MD of the mf103 pattern.

In POAG in the central 20°, the mf103 pattern showed a significant association between mT and MD (negative) as well as MS (positive) (*p* < 0.004). There was no significant association between mT and MS or MD of the complete G2 field.

Neither AP protocol was significantly associated with GCIPL thickness.

**Figure 7:**
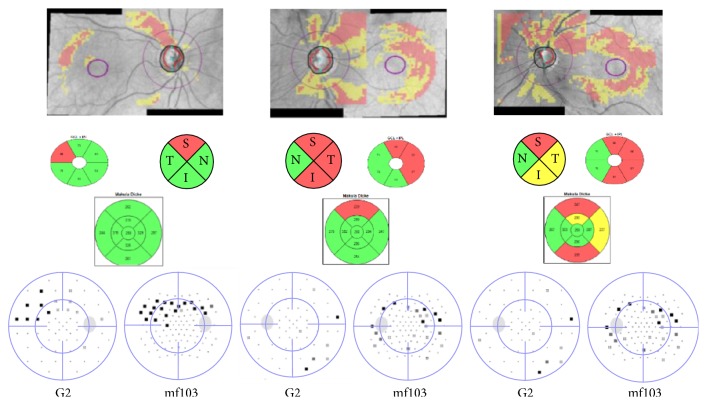
Graphic examples from 3 POAG patients showing the improved correlation between nerve-fiber-bundle defects and visual field parameters for the mf103 pattern compared to the G2 pattern. The top row is a composite of the RNFL and the GCIPL thickness deviation map taken from the Cirrus report. The middle row depicts significant deviations from normal (yellow and red sectors) for the RNFL (upper left), the GCIPL (upper right), and mT (bottom). The lower row shows the corresponding deviation maps for the G2 and the mf103 pattern. It is clear that the mf103 pattern has a higher resolution in the center where the G pattern under samples this field.

Figure 7 shows examples from individual patients. In these patients without central field defects, visual comparison demonstrates the superior correlation of the mf103 pattern to the RNFL thinning when compared to the G2.

### 3.2. Follow-Up

**Figure 8:**
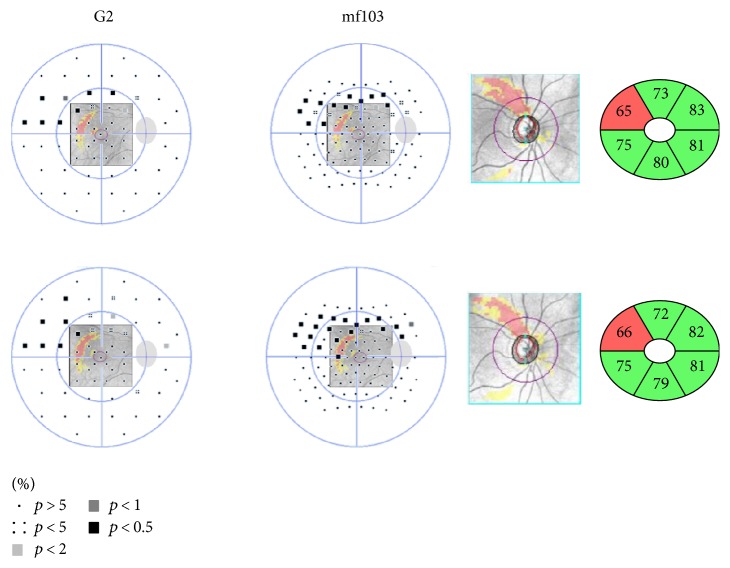
For the first patient in Figure 6, Figure 7 compares the findings at baseline (top) to the one-year follow-up (bottom). The left column shows the G2 pattern and the 2nd column, the mf103 pattern deviation plots as a composite with the GCIPL deviation map. The 3rd column shows the RNFL deviation map and the rightmost column, the GCIPL thickness map as taken from the Cirrus analysis.

Nine POAG patients agreed to the follow-up evaluation at one year.  Figure 8 shows the example of a patient at baseline and at one year. The 103mf pattern shows a clearer and consistent delineation of the arcuate field defect than the G2, where the second visual field obtained at one year might also be mistaken for a quadrant defect. Nonetheless, Table 6 shows that over the one year period, no significant changes had occurred in the glaucoma parameters: MD, MS, and sLV, IOP, mT, and GCIPL. The significant correlation between G2 and mf103 remained (*p* < 0.001).

## 4. Discussion

This study validates a customized visual field protocol (mf103 pattern) based on a multifocal electroretinogram (103 hexagons) pattern, for the assessment of patients with glaucoma.

The mf103 was comparable to the G2 in differentiating patients from controls. The slightly higher MD seen in the mf103 pattern might be caused by better coverage of the paracentral area and a higher frequency of affected test locations in that area. The sample size included in this study was not very large, and therefore a difference of 0.46 dB needs to be interpreted with care. It is however not due to an increased risk of false positives: The risk of more false positives (lower specificity) is not given, if, for example, the number of test locations that have to be at a certain probability level to consider the visual field as abnormal is calculated according to the number of test locations. For example, for a G visual field to be considered abnormal, one needs >59 × 0.05 = 3 test locations at a 5% probability level and for the mf103, one needs >103 × 0.05 = 5 test locations at a 5% probability level. If this is considered, as done here, then sensitivity is not at the price of specificity.

OCT measures (mT, GCIPL, and RNFL) were significantly lower in our POAG patients. In our patient group, only the mf103 pattern showed a meaningful significant relation to mT where MD was increased with thinner mT. The reverse was true for the G2 pattern. We cannot fully explain this discrepancy. One hypothesis could be the differences between SAP patterns in the location and the amount of testing points within the same tested area. Another influencing factor could be the variability in location of the SAP defect in our POAG population. Nevertheless, mT is outperformed by RNFL and GCIPL in glaucoma diagnostic, as conflicting results are observed, depending on stage of disease and type of glaucoma as reviewed by Wong et al. [31].

Surprisingly, neither SAP protocol correlated with GCIPL changes which may reflect the early stages of glaucoma tested, as observed by Lee et al. [32].

In regard to the RNFL, the significant relationship between RNFL sectors and SAP sensitivity in glaucoma based on the Hogan nerve fiber model has been already observed by Naghizadeh et al. [30, 33]. In our study, we observed an improvement in this relationship when applying the mf103 pattern. Thus, the mf103 pattern may be a better tool in glaucoma diagnosis, as the RNFL is supposed to be the most sensitive parameter in glaucoma diagnosis followed by GCIPL and mT [34].

In view of the increasing number of studies which use mfERG in glaucoma to detect glaucomatous dysfunction [15–18, 20, 21, 24–27, 35–37], the mf103 pattern is not just better in regard to structure-function correlation but also as a possibility of having a one-to-one direct correlation between visual field sensitivity and mfERG.

The mf103 pattern is based on the stimulus grid of the 103 hexagon mfERG. In view of structure-function analysis, more test points are included when the mf103 pattern is applied. In individual patients, this appears to better delineate arcuate defects (Figures 6 and 7) and overall, the mf103 pattern correlated better with RNFL thickness than the G2 pattern. This is in agreement with other studies which were able to identify visual field defects beyond the standard resolution from conventional perimetry in glaucoma, applying high spatial resolution perimetry [38–40]. In the nine patients that agreed to follow-up at one year, there was no significant difference in the changes observed in either field.

In this study, the mf103 pattern was programmed on the Octopus Perimeter 900 to exactly reflect the Veris multifocal ERG-103 hexagons scaling. Therefore, the results are not directly comparable to other patterns and other manufacturer ERG devices. For those, the test would have to be reprogrammed according to manufacturer's scaling. If different patterns with different resolutions are applied, the resulting comparison to the G2 pattern needs to be evaluated, as it may differ.

In conclusion, G2 and mf103 pattern can both differentiate patients from controls with no significant difference in performance. The mf103 pattern was more sensitive in defect discrimination and in correlation to RNFL. Thus, the mf103 pattern can be applied in structure and function studies that include the mfERG without compromising sensitivity of the field examination.

## Figures and Tables

**Table 1:** Participants' demographic and clinical variables.

Group	POAG (*n* = 18)	Controls (*n* = 15)	*p* value^∗^
Age (years)	59.6 ± 13.5	49.2 ± 7.2	*p* = 0.012
Sex (M/F)	13/5	4/11	
BCVA (decimal)	0.9 ± 0.0	1.1 ± 0.1	*p* = 0.001
IOP (mmHg)	12.5 ± 1.9	13.2 ± 2.7	*p* = 0.403
C/D ratio	0.7 ± 0.1	0.2 ± 0.0	*p* = 0.000
Duration (min)			
G2	5.6 ± 2.0	5.1 ± 1.3	*p* = 0.436
mf103	8.5 ± 1.7	8.4 ± 2.6	*p* = 0.924
G2 × mf103	3.09 (±2.2)		*p* < 0.01

SD: standard deviation; BCVA: best-corrected visual acuity; IOP: intraocular pressure, if needed under use of topical medication; CDR: cup-to-disc ratio; ^∗^Lavene's Test for equality of variances.

**Table 2:** Overview of the overall OCT measures (*μ*m) for POAG patients and controls.

Group	mT 10	mT 20	GCIPL	RNFL
POAG (*n* = 18)	297.2 ± 13.2	283.6 ± 14.3	67.9 ± 8.9	69.06 ± 11.4
Controls (*n* = 15)	307.5 ± 12.4	293.7 ± 12.4	80.9 ± 4.8	90.27 ± 10.7
POAG versus controls	*p* = 0.029	*p* = 0.0013	*p* < 0.001	<0.001

mT: macula thickness from the central 10° (0°–5° eccentricity) and the central 20° (°0–10° eccentricity); GCIPL: ganglion cell-inner plexiform layer (0°–7.5° eccentricity) and RNFL: retinal nerve fiber layer thickness.

**Table 3:** In this comparison of the G2 and the mf103 pattern, the overall means and standard deviation (±SD) values from both visual field patterns (dB) are given for POAG and controls.

Group	MD		MD103^∗^	MS		MS103^∗^	sLV		sLV103^∗^
POAG (*n* = 18)	3.47 ± 4.0	*p* = 0.136	3.95 ± 3.8	23.3 ± 4.2	*p* = 0.297	23.6 ± 4.0	4.6 ± 2.7	*p* = 0.536	4.8 ± 2.9
Controls (*n* = 15)	0.13 ± 1.7	*p* = 0.138	0.59 ± 1.5	27.2 ± 1.8	*p* = 0.310	27.5 ± 1.6	2.0 ± 0.5	*p* = 0.444	2.2 ± 0.6
POAG versus controls	*p* = 0.0166		*p* = 0.0026	*p* = 0.0152		*p* = 0.0021	*p* < 0.001		*p* < 0.001

For the G2 pattern, MD: mean defect; MS: mean sensitivity; and sLV: squared loss of variance; for the mf103 pattern, MD, MS, and sLV are marked with 103^∗^. *p* values are adjusted to age and gender. Italicized *p* values compare the different field patterns, and upright *p* values compare between POAG and control for each individual pattern.

**Table 4:** Area under the ROC curve (AUC) values of the G2 and the mf103 pattern. The *p* values given in the right column compare the performance between the two field patterns applying the DeLong test.

	AUC	*p* value
MD	0.837	0.878
MD103	0.844	
MS	0.937	0.092
MS103	0.856	
sLV	0.941	0.104
sLV103	0.837	

For the G2 pattern, MD: mean defect; MS: mean sensitivity; and sLV: squared loss of variance; for the mf103 pattern, MD, MS, and sLV are marked with 103.

**Table 5:** The relationship between OCT parameters and field parameters is summarized. Regression coefficients from the linear mixed effects analysis are given. The level of significance is depicted by “^∗^” (^∗^*p* < 0.01 and ^∗∗^*p* < 0.001).

OCT	MD	MD103	MS	MS103
RNFL				
Average	0.88^∗^	0.27	−1.01^∗^	−0.67
Superior	−0.21	−0.37^∗∗^	0.22	0.37^∗^
Nasal	0.42	−0.10	−0.44	0.08
Inferior	−0.52^∗∗^	−0.77^∗∗^	0.56^∗∗^	0.81^∗∗^
Temporal	0.10	−0.64^∗^	−0.13	0.63^∗^
mT				
10°	0.53^∗^	0.17	−0.52^∗^	−0.18
20°	−0.19	−0.35^∗^	0.19	0.35^∗^
GCIPL	0.02	−0.21	−0.02	0.21

For the G2 pattern, MD: mean defect and MS: mean sensitivity; for the mf103 pattern, MD and MS are marked with 103. OCT: RNFL: retinal nerve fiber layer thickness; mT: macula thickness from the central 10° (0°–5° eccentricity), the central 20° (°0–10° eccentricity), and GCIPL: ganglion cell-inner plexiform layer (0°–7.5° eccentricity).

**Table 6:** Summary of the changes observed for the nine patients with a follow-up (FU) at 1 year. Changes in the means and standard deviation (SD) values are given for visual field parameters (dB) and OCT average thickness (*μ*) and also for IOP.

MD	MD103	MS	MS103	sLV	sLV103	IOP	mT	GCIPL
↑ 0.02 ± 1.7	↓ 0.02 ± 2.7	↓ 0.08 ± 1.7	↑ 0.01 ± 2.7	↑ 0.06 0.7	↑ 0.5 1.3	↓ 0.33 ± 2.5	↓ 1.31 ± 7.2	↓ 0.35 ± 0.6
*p* = 0.970	*p* = 0.981	*p* = 0.884	*p* = 0.991	*p* = 0.825	*p* = 0.286	*p* = 0.705	*p* = 0.603	*p* = 0.167

IOP: intraocular pressure in mmHg; For the G2 pattern, MD: mean defect; MS: mean sensitivity; for the mf103 pattern, MD and MS are marked with 103. mT: macula thickness; GCIPL: ganglion cell-inner plexiform layer thickness. ↑ marks an increase and ↓ marks a decrease at 1 year FU.
